# A Hybrid Deep Learning Approach for Bearing Fault Diagnosis Using Continuous Wavelet Transform and Attention-Enhanced Spatiotemporal Feature Extraction

**DOI:** 10.3390/s25092712

**Published:** 2025-04-25

**Authors:** Muhammad Farooq Siddique, Faisal Saleem, Muhammad Umar, Cheol Hong Kim, Jong-Myon Kim

**Affiliations:** 1 Department of Electrical, Electronic and Computer Engineering, University of Ulsan, Ulsan 44610, Republic of Korea; mfarooq229@mail.ulsan.ac.kr (M.F.S.); faisal1999@mail.ulsan.ac.kr (F.S.); muhammadumar@mail.ulsan.ac.kr (M.U.); 2 School of Computer Science and Engineering, Soongsil University, Seoul 06978, Republic of Korea; cheolhong@ssu.ac.kr

**Keywords:** fault diagnosis, continuous wavelet transform, multi-head self-attention, bidirectional long short-term memory, 1D convolutional residual network

## Abstract

This study presents a hybrid deep learning approach for bearing fault diagnosis that integrates continuous wavelet transform (CWT) with an attention-enhanced spatiotemporal feature extraction framework. The model combines time-frequency domain analysis using CWT with a classification architecture comprising multi-head self-attention (MHSA), bidirectional long short-term memory (BiLSTM), and a 1D convolutional residual network (1D conv ResNet). This architecture effectively captures both spatial and temporal dependencies, enhances noise resilience, and extracts discriminative features from nonstationary and nonlinear vibration signals. The model is initially trained on a controlled laboratory bearing dataset and further validated on real and artificial subsets of the Paderborn bearing dataset, demonstrating strong generalization across diverse fault conditions. t-SNE visualizations confirm clear separability between fault categories, supporting the model’s capability for precise and reliable feature learning and strong potential for real-time predictive maintenance in complex industrial environments.

## 1. Introduction

Rotary machines are fundamental to modern industry, playing an essential role in sectors such as manufacturing, aerospace, transportation, and power generation [1,2]. Ensuring their reliable operation is essential to prevent unexpected failures, reduce downtime, and avoid substantial economic losses. Among the key components of rotary machines, rolling bearings are particularly critical as they support rotational motion and maintain mechanical stability [3,4]. However, bearings are also among the most failure-prone elements, responsible for nearly 50% of mechanical breakdowns [5]. Common causes of bearing faults include electrical stress, unbalanced loads, material fatigue, and overloading, which can result in secondary damage and unplanned shutdowns [6,7]. As such, bearing fault diagnosis (BFD) is vital for maintaining operational safety and extending machinery lifespan. A core aspect of BFD is fault feature extraction, which involves analyzing vibration signals collected by sensors through effective signal processing methods to detect fault-related patterns [8]. Although recent advancements in intelligent diagnostic techniques have significantly improved fault detection accuracy, challenges remain, particularly in noisy industrial environments where signal clarity is compromised [9]. Therefore, the development of robust and noise-resilient fault diagnosis methods continues to be a crucial area of research in industrial health monitoring [10,11,12].

Traditional approaches to bearing fault diagnosis rely on model-based, empirical, or machine learning (ML)-driven methods. Model-based techniques use mathematical equations to simulate and predict bearing behavior, whereas empirical methods depend on domain expertise and manual analysis of vibrational or acoustic emission signals [13,14]. However, the growing complexity of modern machinery has made it increasingly difficult to develop accurate models and rely solely on human expertise. Additionally, these traditional methods often struggle to adapt to dynamic operating conditions, varying fault severities, and noise-prone environments. They also require extensive feature engineering, which is time-consuming and prone to human bias. To overcome these limitations, data-driven methods, particularly those based on deep learning (DL) have gained significant traction in fault detection and classification due to their ability to automatically learn complex patterns directly from raw sensor data [15,16,17].

In vibration signal-based fault diagnosis, various machine learning algorithms, such as support vector machine (SVM), random forest, and K-nearest neighbors (KNN), have been widely used [18,19,20]. These methods involve multiple steps, including signal pre-processing, feature extraction, and classification. However, traditional ML-based approaches often depend heavily on manually crafted features, limiting their generalizability and efficiency in real-world scenarios. To address these challenges, deep learning methods, particularly convolutional neural networks (CNNs) and long short-term memory (LSTM) networks, have emerged as powerful alternatives. CNNs have demonstrated remarkable ability in extracting spatial patterns from vibration signals, while BiLSTM models excel at capturing temporal dependencies in time-series data [21].

Deeper DL architectures further enhance performance by capturing complex patterns; however, they often encounter gradient-related issues like vanishing or exploding gradients that hinder effective training and reduce diagnostic accuracy. To mitigate these problems, He et al. [22] introduced the residual network (ResNet), which employs shortcut connections to maintain gradient flow during backpropagation. Building on this, Zhang et al. [23] proposed an attention-augmented ResNet for fault diagnosis in wind-turbine gearboxes, which effectively captured time-frequency features and enhanced classification performance. Similarly, Liang et al. [24] used wavelet transformation in combination with an improved ResNet for bearing fault detection, while Zhao et al. [25] introduced a deep residual shrinkage network that further improved diagnostic precision. These innovations demonstrate the advantages of residual learning frameworks like ResNet over traditional CNN architectures in fault diagnosis tasks. Despite the notable progress in deep learning-based fault diagnosis, current models still face challenges in effectively capturing both spatial and temporal features, especially in nonstationary and noisy industrial environments. Standard CNNs often struggle to focus on the most relevant regions of complex signals, while LSTM-based models alone may not fully exploit local feature hierarchies or suppress irrelevant noise [21]. Additionally, many existing approaches lack the adaptability needed to generalize across varying fault conditions and datasets [26]. These limitations highlight the need for a more comprehensive and robust diagnostic framework capable of extracting discriminative features, handling temporal dependencies, and enhancing attention to critical signal components under real-world conditions [27].

This study builds upon these advancements by proposing a novel hybrid deep learning approach for bearing fault diagnosis. The proposed model integrates CWT with a deep learning framework combining MHSA, BiLSTM, and 1D conv ResNet. This integration enhances feature extraction, suppresses noise, and improves the model’s ability to learn temporal dependencies from nonstationary and nonlinear vibrational signals. Unlike conventional CNNs, the inclusion of MHSA allows the model to focus on critical signal regions, while BiLSTM captures long-term dependencies essential for accurate fault identification. The effectiveness of the proposed model is evaluated using both a controlled laboratory bearing dataset and the publicly available Paderborn bearing dataset to assess its generalizability across different fault conditions. The model achieves superior classification performance, demonstrating high precision, recall, and robustness in distinguishing fault types. The results confirm the model’s suitability for real-time predictive maintenance, offering an efficient and reliable solution for intelligent fault detection in industrial applications. The key innovations and contributions of this study are as follows:

(1) Hybrid Deep Learning Architecture: A novel deep learning framework is introduced, combining 1D conv ResNet, MHSA, and BiLSTM. This hybrid approach enhances feature extraction, suppresses noise, and captures long-range dependencies in nonstationary and nonlinear signals.
(2) Improved Fault Classification Performance: Unlike conventional backpropagation-based models, the proposed architecture efficiently processes time-series data with enhanced feature learning and temporal modelling, leading to superior classification accuracy and robustness.
(3) Comprehensive Validation on Multiple Datasets: The model was validated on both a bearing testbed dataset and the Paderborn dataset, demonstrating its generalizability and effectiveness in diverse fault conditions.
(4) Enhanced Interpretability and Reliability: The experimental results confirm the model’s ability to accurately distinguish fault types, with t-SNE visualizations showcasing clear separability between fault classes, making it a viable solution for real-time predictive maintenance in industrial applications.

The remainder of this paper is structured as follows:

- Section 2 details the proposed methodology, including data preprocessing, feature extraction, and model architecture;
- Section 3 presents the experimental setup, results, and performance analysis;
- Section 4 provides the conclusion and potential future directions.

## 2. Proposed Method for Fault Diagnosis in Bearings

**Figure 1:**
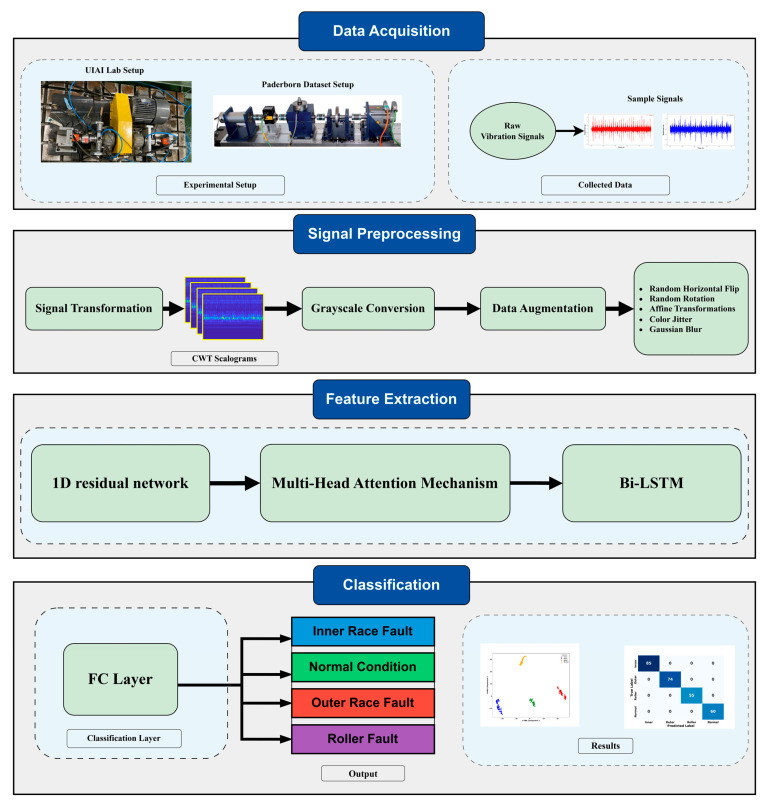
Workflow of the proposed hybrid CWT-based deep learning model integrating MHSA, BiLSTM, and 1D conv ResNet for bearing fault diagnosis.

The proposed methodology integrates CWT for time-frequency analysis with a hybrid deep learning model combining MHSA, BiLSTM, and 1D conv ResNet in the following four steps as shown in Figure 1.

Step 1—Data Acquisition:

Vibration signals are collected from two setups: the UIAI Lab experimental platform and the Paderborn bearing test rig. These signals, representing different bearing fault conditions, are acquired using accelerometers and AE sensors to ensure high-quality data for analysis.

Step 2—Signal Preprocessing:

The raw signals are first transformed into time-frequency representations using CWT, producing scalograms. These scalograms are then converted to grayscale and subjected to data augmentation techniques (e.g., flipping, rotation, affine transformation, color jitter, and Gaussian blur) to enhance generalization and reduce class imbalance.

Step 3—Feature Extraction:

The preprocessed scalograms are fed into a hybrid deep learning framework. A 1D residual network extracts spatial features, which are refined using a MHSA mechanism to focus on critical fault regions. The BiLSTM layer then captures temporal dependencies, ensuring comprehensive feature representation.

Step 4—Classification:

The refined features are passed through FC layers with softmax activation to classify the signal into one of four categories: inner race fault, outer race fault, roller fault, or normal condition (i.e., IRF, ORF, RF, NC respectively). Results are visualized using metrics such as confusion matrices, t-SNE plots and ROC curves to confirm classification performance and fault separability.

### 2.1. Continuous Wavelet Transform

CWT is a fundamental technique in analyzing bearing vibration signals, especially for detecting and classifying faults in rolling element bearings. Defects in bearing components generate transient and non-stationary vibrations, which challenge traditional techniques like Fourier transform and short-time Fourier transform due to their assumptions of stationarity and fixed window sizes [28]. CWT overcomes these by offering a time-frequency representation, allowing precise fault signature identification [29]. Mathematically, the CWT is defined as a convolution of the signal $x\left(t\right)$ with a scaled and translated wavelet function *ψ* (*t*), given by.

(1) $$C\left(a,b\right)=\int_{-\infty }^{\infty }x\left(t\right)\frac{1}{\sqrt{a}}\psi \left(\frac{t-b}{a}\right)dt$$

where a represents the scale factor, which determines the frequency resolution, and b denotes the translation, which controls the time localization of the transformation. The mother wavelet $\psi \left(t\right)$ serves as a basis function, affecting the sensitivity of CWT to fault-related features. A larger a highlights low-frequency components, while a smaller a captures high-frequency transients. This multi-resolution nature is ideal for detecting localized impulses from defects [30]. The scalogram defined as,

(2) $$S\left(a,b\right)=\left|{\left.C\left(a,b\right)\right|}^{2}\right.$$

Equation (2) provides a visual representation of how energy is distributed across time and frequency. The presence of localized high-energy regions in the scalogram often indicates the occurrence of periodic fault impacts. Adaptive filtering may suppress fault-related high frequencies [31]. EMD suffers from mode mixing, embedding noise in fault features [32]. CWT provides an effective solution by distributing noise energy across multiple scales, reducing its impact on fault detection while preserving transient fault signatures. Another key aspect of CWT in bearing fault diagnosis is its ability to detect fault frequencies associated with defects in different bearing components can be mathematically expressed as,

(3) $$f_{bpf}=\frac{n}{2}f_{r}(1+\frac{D}{d}\cos\theta )$$

(4) $$f_{bpfo}=\frac{n}{2}f_{r}(1-\frac{D}{d}\cos\theta )$$

where $f_{bpf}$ and $f_{bpfo}$ correspond to the fault frequencies of the inner and outer races, respectively, n is the number of rolling elements, $f_{r}$ is the shaft rotational frequency, D is the pitch diameter, d is the rolling element diameter, and θ is the contact angle. These frequencies appear as periodic bursts in scalograms with unique energy patterns. Wavelet choice influences CWT’s performance. The Morlet wavelet, resembling a sinusoid modulated by a Gaussian, is ideal for frequency analysis. Selecting wavelets involves balancing time and frequency resolution to capture transients and minimize noise. A key feature extraction method enabled by CWT is the calculation of wavelet energy entropy, which quantifies the complexity of the time-frequency representation and can serve as an indicator of fault severity. The wavelet energy entropy is defined as

(5) $$E=-\sum_{i=1}^{n}p_{i}\log p_{i}$$

where $p_{i}$ is normalized wavelet energy in each band. Higher entropy implies complex, multi-frequency signals; lower entropy indicates regular patterns, possibly from well-defined faults. This helps distinguish fault types and track damage severity. CWT transforms vibration signals into a domain where fault characteristics are clear. Its scalograms carry rich diagnostic information for precise fault localization and severity estimation. By capturing transient and stationary components, CWT offers better interpretability over conventional time and frequency-domain methods [33].

### 2.2. 1D Residual Network

A 1D ResNet is an advanced deep learning architecture designed for sequential one-dimensional data, making it well-suited for vibration signals, time-series analysis, and acoustic emissions. In the proposed model, it extracts meaningful features from raw vibration signals before passing them to attention and BiLSTM layers. Its residual learning framework enables deep feature extraction while avoiding the vanishing gradient problem. The core unit of a 1D ResNet is the residual block, comprising convolutional layers, batch normalization, and activation functions [34]. Rather than learning a new representation, the residual block models the difference between input and output:

(6) $$H\left(x\right)=F\left(x\right)+x$$

where x is the input signal, $F\left(x\right)$ represents the learned transformation through a series of convolutions, and $H\left(x\right)$ is the final output. The presence of the identity mapping x ensures that essential signal characteristics are preserved, preventing excessive modifications that could affect fault-related features in vibration signals. Each block applies multiple 1D convolutions sliding over time to extract local and global patterns. The convolution operation is defined as:

(7) $$y_{i}=\sum_{k=1}^{K}w_{k}\cdot x_{i+k}+b$$

where $y_{i}$ is at the position i, $w_{k}$ are learnable weights, K is kernel size, and b is bias. After each convolution operation, batch normalization is applied to normalize the feature distribution and stabilize training. This normalization is computed as:

(8) $$\hat{x}=\frac{x-u}{\sigma }$$

where u is the batch mean and σ is the batch standard deviation. This ensures that the network maintains stable feature distributions across layers, accelerating convergence and improving generalization. The network then applies a ReLU (rectified linear unit) activation function, which introduces nonlinearity into the model, allowing it to learn complex representations. ReLU is defined as:

(9) $$f\left(x\right)=\max\;\left(0,x\right)$$

**Figure 2:**
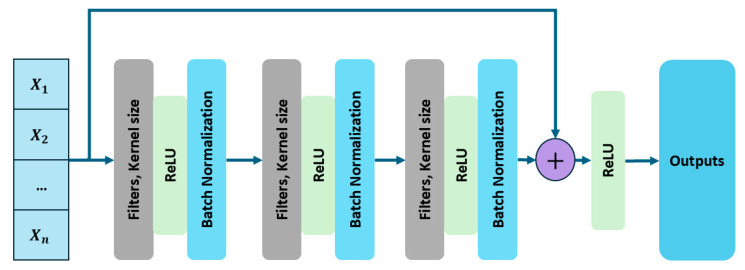
Architecture of the 1D convolutional residual network used for extracting deep temporal-spatial features from CWT-based vibration data.

Which ensures that only positive activations are passed forward, preventing issues such as vanishing gradients. The residual connection then adds the original input signal x back to the transformed features $f\left(x\right)$, ensuring that important signal characteristics are not lost during deep feature extraction. As the vibration signal passes through the ResNet, early layers extract statistical features while deeper layers extract high-level representations as shown in Figure 2. These features enable precise classification of bearing faults. Mathematically, in a residual network with L layers, the transformation applied to the input signal can be expressed as:

(10) $$H_{L}\left(x\right)=x+\sum_{i=1}^{L}F_{i}(x)$$

where $F_{i}(x)$ represents the feature transformation learned at the $i^{th}$ residual block. The presence of the original input x in the final output ensures that even if some layers contribute only minor refinements, the core structure of the signal remains intact. The 1D ResNet has three convolutional layers, each with batch normalization and ReLU, ensuring multi-scale signal variation capture while avoiding overfitting. The residual output is passed to a multi-head attention mechanism, which enhances relevant features and suppresses noise. This refinement is essential because vibration signals often contain background interference. Integrating 1D ResNet ensures deep hierarchical feature learning without performance degradation. Its ability to extract time- and frequency-domain features suits real-world, non-stationary vibration signals.

### 2.3. Multi-Head Self-Attention Mechanism

The MHSA mechanism is an important component in modern deep learning architectures, especially for sequential data like vibration signals, NLP, and fault diagnosis [35]. In the proposed model, MHSA enhances feature representations from the 1D ResNet by selectively focusing on different parts of the extracted feature maps. It captures long-range dependencies in time-series data, essential for analyzing extended fault-induced patterns. At the core of MHSA is self-attention, which assigns dynamic importance to different positions in the input. Unlike convolutional operations with local receptive fields, attention mechanisms compute global dependencies across all time steps. This is useful in bearing fault diagnosis, where defects create periodic or irregular patterns not always captured by convolution alone. Mathematically, self-attention is computed using three key transformations: the query (*Q*), key (*K*), and value (*V*) matrices. Given an input sequence represented as a matrix X, these matrices are obtained through learned weight matrices:

(11) $$Q=XW^{Q},K=XW^{K},V=XW^{V}$$

where $W^{Q}$, $W^{K}$, and $W^{V}$ are trainable weight matrices that project the input sequence into different sub-spaces. The attention scores are computed using the scaled dot-product attention formula

(12) $$Attention\left(Q,K,V\right)=softmax\left(\frac{QK^{T}}{\sqrt{d_{k}}}\right)V$$

where $d_{k}$ is the dimension of the key vectors, and the softmax function ensures that the attention weights sum to one, allowing the model to focus more on important regions. The division by $\sqrt{d_{k}}$ prevents the dot product values from becoming excessively large. The multi-head attention mechanism extends this concept by performing multiple attention operations in parallel, each with a different set of learned weights. The input is projected into multiple lower-dimensional subspaces, each producing its own attention output:

(13) $$MultiHead\left(X\right)=Concat\left({head}_{1},{head}_{2},\ldots ,{head}_{h}\right)W^{O}$$

where each attention head is computed independently as

(14) $${head}_{i}=Attention\left({QW}_{i}^{Q},{KW}_{i}^{K},{VW}_{i}^{V}\right)$$

The final output is obtained by concatenated and linearly transformed by $W^{O}$. This allows analysis from multiple perspectives: some heads may attend to high-frequency transients, others to low-frequency trends, creating a richer representation. In bearing fault diagnosis, MHSA refines 1D ResNet features. While ResNet captures local dependencies, it lacks global correlation. Attention is compensated by reweighing feature maps, emphasizing fault-relevant patterns [36].

**Figure 3:**
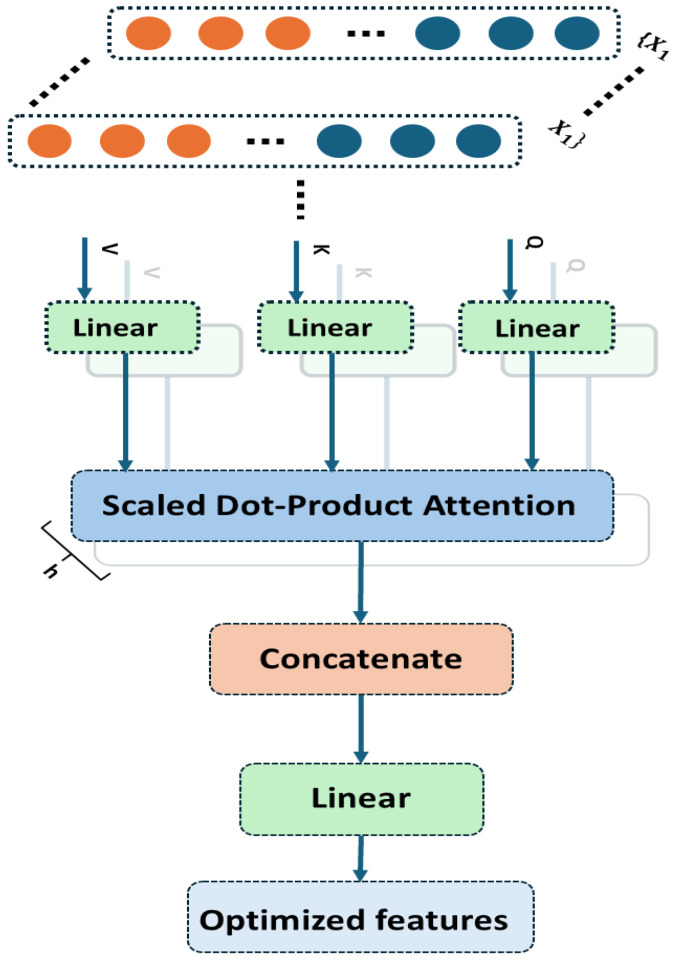
Illustration of the multi-head self-attention mechanism used to enhance feature representation by capturing interdependence in time-frequency features.

Figure 3 illustrates the inner workings of the MHSA mechanism. The input features are transformed into *Q*, *K*, and *V* vectors through learned linear projections. Each attention head computes scaled dot-product attention independently, capturing different contextual dependencies. These parallel outputs are concatenated and passed through a final linear transformation, enabling the network to focus on multiple fault-relevant regions in the signal concurrently. By integrating MHSA, the model becomes more robust to variations in load, speed, and operating conditions. Each head captures a distinct representation, improving generalization across fault types. The attention-enhanced features are then passed to the BiLSTM layer, where temporal dependencies are further processed before classification.

### 2.4. Bidirectional Long Short-Term Memory

A BiLSTM network is an advanced deep learning architecture designed to capture both past and future dependencies in sequential data, making it highly effective for time-series analysis, speech recognition, and fault diagnosis [37]. In bearing fault detection, a BiLSTM models long-term dependencies in vibration signals, enabling accurate fault classification. Traditional RNNs suffer from vanishing gradients, limiting their ability to learn long sequences. LSTM overcomes this using gating mechanisms that control information flow, retaining relevant features while discarding noise [38].

The core component of an LSTM is the memory cell, which maintains long-term dependencies across time steps. This memory cell is controlled by three main gates: the forget gate $f_{t}$, which decides how much of the previous cell state $C_{t-1}$ should be retained or forgotten; the input gate $i_{t}$, which determines how much new information ${\tilde{C}}_{t}$ should be added to the memory; and the output gate $o_{t}$, which controls how much of the updated memory should be passed forward as the hidden state. These gates are computed as follows:

(15) $$f_{t}=\sigma (W_{f}\left[h_{t-1},X_{t}\right]+b_{f})$$

(16) $$i_{t}=\sigma (W_{i}\left[h_{t-1},X_{t}\right]+b_{i})$$

(17) $${\tilde{C}}_{t}=tanh(W_{C}\left[h_{t-1},X_{t}\right]+b_{C})$$

(18) $$C_{t}=f_{t}⊙C_{t-1}+i_{t}⊙{\tilde{C}}_{t}$$

(19) $$o_{t}=\sigma (W_{o}\left[h_{t-1},X_{t}\right]+b_{o})$$

(20) $$h_{t}=o_{t}⊙tanh(C_{t})$$

where σ represents the sigmoid activation function, which ensures that values are between 0 and 1, and tanh introduces nonlinearity to help model complex sequential relationships. The element-wise multiplication ⊙ allows for selective updates to the memory and hidden states, ensuring that only the most relevant information is retained. Unlike a traditional LSTM, which only processes the input sequence in one direction (from past to future), a BiLSTM extends this by introducing two LSTM networks, one moving forward and the other moving backward through time. This bidirectional structure allows the model to capture dependencies in both directions, making it particularly useful in tasks where context from both past and future time steps is essential. The forward LSTM computes

(21) $$\vec{h_{t}}=LSTM\left(X_{t},\vec{h_{t-1}}\right)$$

while the backward LSTM processes the sequence in reverse:

(22) $$\overset{\leftarrow }{h_{t}}=LSTM\left(X_{t},\overset{\leftarrow }{h_{t+1}}\right)$$

The final hidden state at each time is obtained by concatenating the outputs of both directions, such as

(23) $$h_{t}=[\vec{h_{i}};\overset{\leftarrow }{h_{t}}]$$

**Figure 4:**
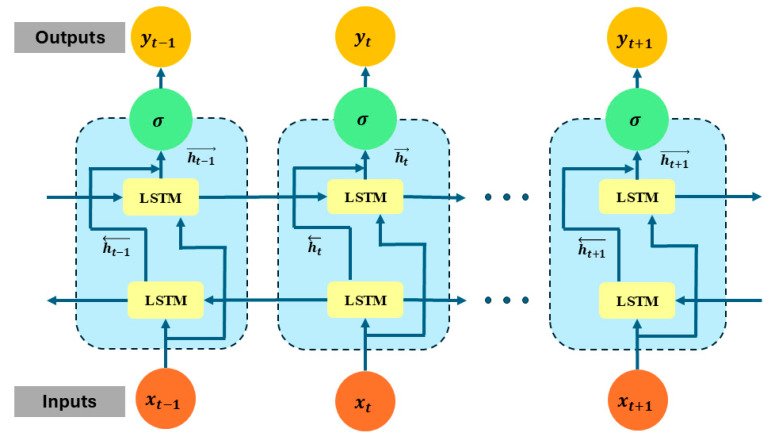
Structure of the bidirectional LSTM module for learning forward and backward temporal patterns in CWT-transform vibration signals.

This bidirectional process significantly improves the model’s ability to capture temporal dependencies in vibration signals, particularly in bearing fault detection, where fault-induced patterns may be spread over time rather than occurring at fixed intervals. In the proposed model, BiLSTM follows the 1D ResNet and MHSA mechanism. ResNet extracts hierarchical features, MHSA enhances them, and BiLSTM models short- and long-term dependencies. This improves discrimination between normal and faulty states. BiLSTM effectively handles varying fault classes. As defects evolve, they alter vibration signal patterns—BiLSTM detect these transitions using both past and future context [39]. It also handles noisy data by learning directly from raw signals, filtering out irrelevant noise and focusing on fault signatures. The output is a sequence of representations encoding both local and long-range fault features. This ensures that the model utilizes both spatial and temporal information, optimizing fault detection accuracy [40]. Mathematically, the FC layer applies a linear transformation followed by a nonlinear activation function. Given an input feature vector h of size d, the fully connected layer computes the output y using the equation,

(24) $$y=W_{h}+b$$

where W represents the weight matrix, which contains the learnable parameters defining how inputs are mapped to outputs, while b is the bias vector, providing additional flexibility to the transformation. Following this transformation, a nonlinear activation function is applied to enable the network to learn complex mappings. For classification tasks, the final FC layer typically employs the softmax activation function, which converts the raw output scores (logits) into a probability distribution over the classes. The softmax function is defined as

(25) $$P\left(y_{i}\right)=\frac{e^{z_{i}}}{\sum_{j=1}^{N}e^{z_{j}}}$$

where $z_{i}$ represents the raw score for class i, N denotes the total number of classes, and $P\left(y_{i}\right)$ represents the probability of the input belonging to class i. To prevent overfitting and enhance generalization, dropout regularization is often applied within FC layers. Dropout functions by randomly deactivating a fraction of neurons during training, ensuring that the network does not become overly reliant on specific neurons and instead learns more generalized representations. Mathematically, dropout is implemented as

(26) $$h^{\prime }=D⊙h$$

where D represents a binary mask with values drawn from a Bernoulli distribution, h is the input feature vector before dropout, and $h^{\prime }$ is the modified feature vector after dropout [41]. The number of fully connected layers in a network varies based on the complexity of the problem. In most classification models, one or two fully connected layers are followed by a final softmax layer, which produces probability distribution over the output classes. The final classification decision is determined by selecting the class with the highest probability, computed as

(27) $$\hat{y}=\arg\max P(y_{i})$$

where $\hat{y}$ represents the predicted fault class. This structured decision-making process ensures that the model outputs a robust and reliable prediction based on the learned representations as depicted in Figure 4.

Overall, the FC layers in the proposed method function as the final stage of the diagnosis pipeline, transforming deep hierarchical representations into a structured probability distribution over fault classes. It is worth mentioning that the three FC layers serve different roles—the first (1024 units) expands the feature space for deep representation learning, the second (512 units) compresses features to reduce complexity and overfitting, and the final (4 units) maps features to fault classes using softmax activation for classification. The proposed model architecture summary and training parameter details are summarized in the following Table 1 and Table 2, respectively.

## 3. Results and Performance Evaluation

The effectiveness of the proposed method is assessed using VS data obtained from actual bearing testbeds. Since the primary aim of this method is to detect and diagnose faults in bearings, this section begins by comparing the fault detection capability of the proposed method.

### 3.1. Experimental Setup and Data Acquisition

**Figure 5:**
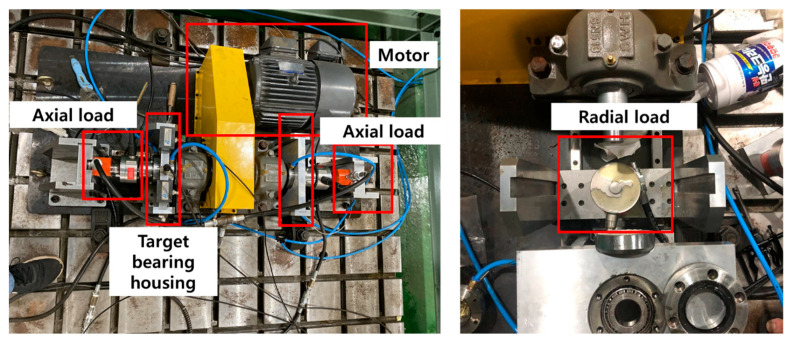
Experimental setup for acquiring vibration signals under various fault conditions in a controlled laboratory environment.

**Figure 6:**
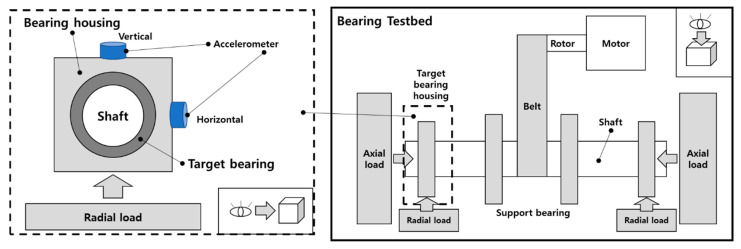
Schematic diagram of experimental setup for bearing fault diagnosis.

Figure 5 depicts the structure of the bearing testbed developed by the Ulsan Industrial Artificial Intelligence (UIAI) Laboratory at Ulsan University, Ulsan, Republic of Korea, which was utilized for this analysis. The collected data were categorized into four distinct bearing conditions: normal, outer race damage, inner race damage, and roller damage. During the experiment, a three-phase motor operated the testbed at a constant speed of 1800 rpm. The rotational motion was transferred from the rotor shaft to the main shaft via a belt system installed on both sides of the test bearings. The bearings used in this study were FAG NJ206-E-TVP2 cylindrical roller bearings made of chrome steel (AISI 52100), subjected to a constant vertical load of 100 kgf. Artificial defects were introduced with dimensions of approximately 3 mm × 0.3 mm × 1 mm to simulate real fault conditions. To ensure precise data acquisition, the maximum vibration signal was recorded from the left side of the target bearing using both a vibration sensor and an acoustic emission (AE) accelerometer. A schematic representation of the complete experimental setup is provided in Figure 6, offering a detailed overview of the testbed configuration and data acquisition process.

The data acquisition system, detailed in Table 3, utilizes a FAG NJ206-3-TVP2 bearing, a cylindrical roller type. For capturing VS, an accelerometer (model PCB-622B01) was employed, while AE signals were detected using R15I-AST AE sensors. Both sensors were interfaced with an NI-9234 data acquisition (DAQ) device to ensure precise data collection from the integrated electronics piezoelectric (IEPE) sensors.

**Figure 7:**
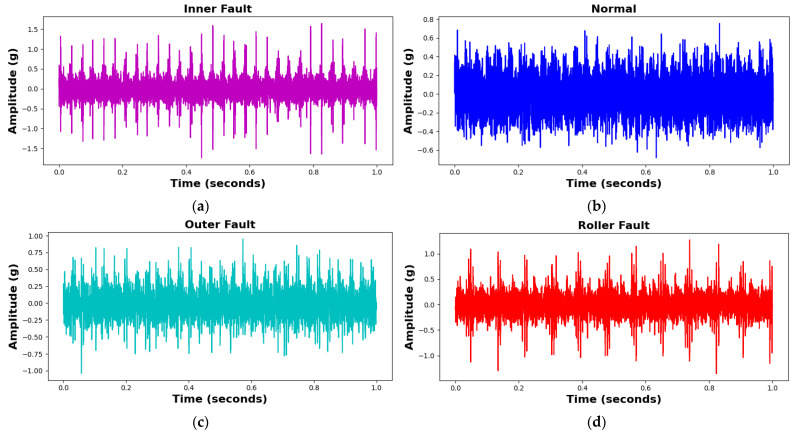
Vibration time domain signals for different bearing fault conditions: (**a**) IRF, (**b**) NC, (**c**) ORF, and (**d**) RF.

**Figure 8:**
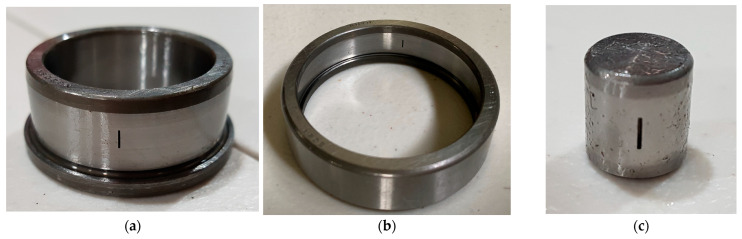
Fault components used during the experiment: (**a**) IRF, (**b**) ORF, and (**c**) RF.

Vibration data were acquired at a sampling rate of 25 kHz, with five minutes of continuous data collection conducted for all bearing conditions. Subsequently, the data were divided into 1 s segments, with each 1 s segment containing 309–390 data samples for different fault types. The test procedure can be repeated for different fault types by replacing the test bearing in the same testbed setup. The samples of each class of the bearing fault as well as its vibration data are shown in the following Figure 7 and Figure 8, followed by the dataset details in Table 4.

### 3.2. Paderborn University Dataset

**Figure 9:**
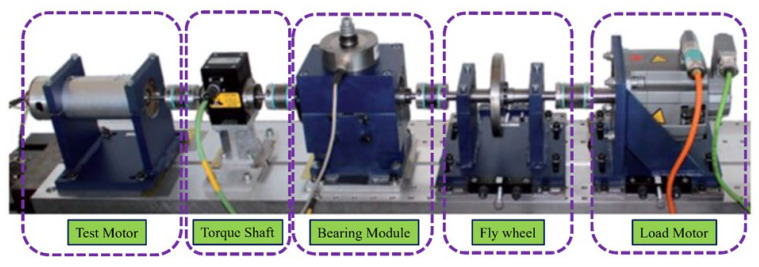
Experimental setup of the Paderborn bearing dataset used for validating the model on real and artificially induced fault scenarios.

The experimental test rig developed by Lessmeier et al. [42] for vibration data acquisition consisted of several key components, as illustrated in Figure 9. Designed as a versatile platform, it enabled the testing of bearings under various fault conditions, allowing for precise fault characterization and analysis. At the core of the setup, the bearing test module facilitated controlled testing by applying a constant radial load to the test bearings, adjustable up to 10 kN before each experiment. This ensured consistency and repeatability across different testing conditions. The system was powered by a 425 W permanent magnet synchronous motor (PMSM) with a nominal torque of 1.35 Nm, a rotational speed of 3000 rpm, a nominal current of 2.3 A, and four pole pairs. Manufactured by Hanning Elektro-Werke GmbH & Co. KG (Model: SD4CDu8S009), the motor was controlled using an industrial inverter (KEB Combivert 07F5E 1D-2B0A, Oerlinghausen, Germany) with a 16 kHz switching frequency, effectively replicating real industrial environments, including PWM-induced noise typically found in such systems. To capture vibration signals, a piezoelectric accelerometer (Model 336C04, PCB Piezotronics, Inc., Depew, NY, USA) was securely mounted on the bearing housing adapter. The signals were amplified using a Type 5015A charge amplifier (Kistler Group, Winterthur, Switzerland) and filtered through a 30 kHz low-pass filter, which removed high-frequency noise. The filtered signals were then digitized at a 64 kHz sampling rate, ensuring high-resolution data acquisition for precise fault analysis. To mimic real-world operational conditions, a flywheel and load motor were incorporated into the setup, simulating dynamic load variations and system inertia. The experiments were conducted under four distinct operating conditions, varying in rotational speed, radial force, and load torque, as outlined in Table 5.

The study utilized 32 bearings, categorized into three groups: 12 bearings with artificially induced faults, 14 bearings subjected to accelerated lifetime testing to develop real-time faults, and 6 healthy bearings serving as baseline references.

#### 3.2.1. Case 1: Artificial Induced Faults

Lessmeier et al. [42] induced artificial damage in bearings using three distinct methods, each designed to replicate common fault characteristics observed in industrial environments. The first method, electric discharge machining (EDM), was employed to create precise trenches approximately 0.25 mm in length along the rolling direction, with depths ranging from 1 mm to 2 mm. This technique ensures highly controlled and repeatable damage patterns, making it ideal for maintaining consistent experimental conditions. The second method involved drilling, which was used to introduce faults of varying diameters, simulating damage caused by localized stress concentrations or surface abrasions. This approach helps replicate real-world wear and tear commonly found in industrial applications. The third method utilized electric engraving to create faults with damage lengths ranging from 1 mm to 4 mm. This technique produces irregular damage profiles, mimicking defects that arise due to material fatigue or improper handling. By employing these three damage-inducing techniques, the dataset captures a diverse range of fault characteristics, ensuring a comprehensive evaluation of the proposed fault detection framework. The bearing codes used in this study for Case 1, which are publicly available, are detailed in Table 6.

#### 3.2.2. Case 2: Real Bearing Faults

Lessmeier et al. [42] generated real damaged ball bearings using an accelerated lifetime test rig. The test bearings were subjected to a radial load applied through a spring-screw mechanism. To accelerate the formation of fatigue damage, the load was deliberately set higher than standard operational conditions while remaining below the bearings’ static load capacity to prevent immediate failure. Additionally, low-viscosity oil was introduced to simulate poor lubrication conditions, further promoting the gradual development of bearing damage. The accelerated testing process produced several damaged bearings, with approximately 70% exhibiting fatigue-induced pitting on both the inner race (IR) and outer race (OR). Among the remaining bearings, most displayed plastic deformations in the form of indentations caused by debris, primarily manifesting as OR faults. One bearing experienced a complete fracture, while no damage was observed to the rolling elements. This controlled approach to accelerated lifetime testing ensured the generation of realistic and diverse fault scenarios, allowing the collected data to closely reflect real-world failure mechanisms. This makes the dataset valuable for robust fault detection research. The bearing codes used in this study for Case 2, which are publicly available, are listed in Table 7.

### 3.3. Performance Metrics for Comparisons

The evaluation of the proposed method was conducted using key performance metrics, including accuracy, precision, recall, and F1-score, as outlined in Equations (28)–(31). These metrics were essential in measuring the model’s ability to accurately classify different fault conditions in the milling machine. By leveraging this comprehensive evaluation approach, the proposed model was thoroughly assessed for its capability in real-world fault detection and diagnosis, ensuring its suitability for industrial applications. The proposed model was validated using stratified k-fold cross validation (k = 5) to avoid overfitting. This thorough evaluation confirmed the model’s reliability and effectiveness in practical fault classification tasks.

(28) $$Accuracy=\frac{\left(TN+TP\right)}{\left(TP+TN+FP+FN\right)}\times 100\%$$

(29) $$Precision=\frac{TP}{TP+FP}\times 100\%$$

(30) $$Recall=\frac{TP}{TP+FN}\times 100\%$$

(31) $$F1-Score=\frac{2TP}{2TP+FP+FN}=\frac{2\times (Precision\times Recall)}{Precision+Recall}$$

True positives (TPs) indicate instances where the model correctly identifies faulty conditions, while true negatives (TNs) refer to non-faulty cases that are accurately recognized. False positives (FPs) occur when the model incorrectly labels non-faulty instances as faulty, whereas false negatives (FNs) represent faulty cases that are misclassified as non-faulty. Together, these metrics provide a comprehensive evaluation of the model’s classification performance, offering valuable insights into its robustness, accuracy, and reliability in detecting and diagnosing bearing faults.

### 3.4. Comparative Analysis of Fault Diagnosis Methods

**Figure 10:**
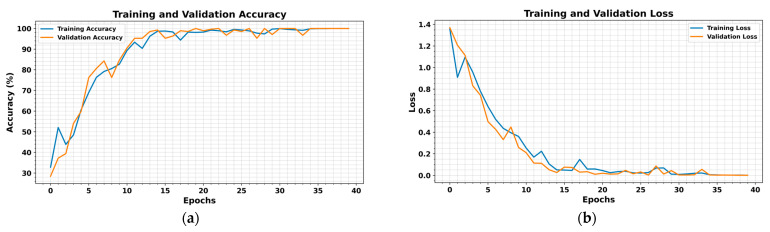
Proposed model training and validation. (**a**) Accuracies vs number of epochs and (**b**) losses vs number of epochs.

**Figure 11:**
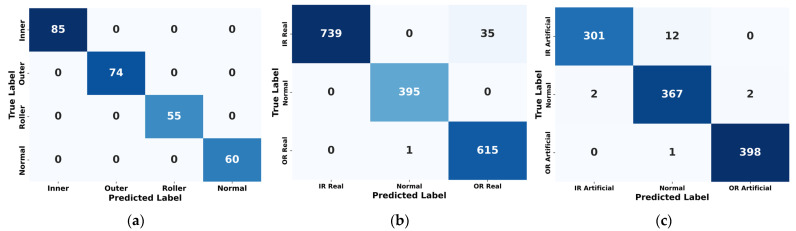
Confusion matrices of the proposed model on (**a**) UIAI, (**b**) Paderborn real, and (**c**) Paderborn artificial datasets, showing classification accuracy across fault classes.

**Figure 12:**
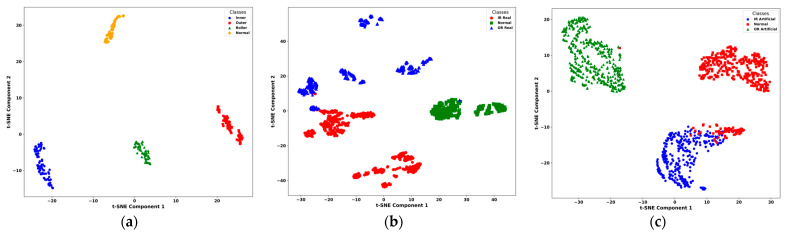
t-SNE visualizations of learned features on (**a**) UIAI, (**b**) Paderborn real, and (**c**) Paderborn artificial datasets, showing clear fault class separation.

To comprehensively evaluate the performance and generalization capability of the proposed model, it was tested on the UIAI Lab dataset as well as both the real and artificial subsets of the Paderborn dataset. Each of the datasets was divided into 80% for training and 20% for testing purposes. Furthermore, the UIAI Lab dataset was also used to validate the recent model proposed by Guanghua Fu et al. [43] for a direct performance comparison, and an ablation study of the proposed model was carried out. The proposed hybrid deep learning model combines CWT with an advanced classification architecture comprising 1D conv ResNet, MHSA, and BiLSTM. Initially, vibration signals are transformed into time-frequency representations using CWT, effectively capturing nonstationary and nonlinear signal characteristics across multiple scales. These CWT scalograms are then processed through the hybrid deep learning network. The proposed hybrid deep learning model combines CWT with a powerful classification framework that integrates MHSA, BiLSTM, and 1D conv ResNet. Initially, vibration signals are transformed into time-frequency representations using CWT, effectively capturing nonstationary and nonlinear signal characteristics across multiple scales. These CWT scalograms are then processed through the hybrid architecture, where each component contributes to robust and interpretable feature learning: 1D conv ResNet extracts localized features while ensuring efficient gradient propagation, BiLSTM captures long-term temporal dependencies important for fault progression tracking, and MHSA enhances the model’s focus on the most informative segments of the signal. This enables the model to remain resilient in noisy industrial environments and effectively distinguish between fault types. The model was trained on a bearing dataset encompassing four classes—IRF, ORF, RF, and NC. Training and validation results demonstrated the model’s rapid convergence and stability. Both accuracy curves quickly approached higher accuracy after a dozen epochs, while training and validation losses showed a sharp decline and stabilize near zero—indicating highly effective learning without overfitting, as shown in Figure 10. On internal testing, the model achieved perfect classification accuracy across all four fault categories, as reflected in the confusion matrix in Figure 11: 85/85 for IRF, 74/74 for ORF, 55/55 for RF, and 60/60 for NC. Precision, recall, and F1-scores for all classes reached 1.00, further emphasizing the model’s diagnostic precision, as shown in Table 8. The t-SNE visualization revealed clearly clustered and non-overlapping distributions for each class, showcasing the model’s ability to learn well-separated, discriminative features as evident from Figure 12. Additionally, ROC curves for each class yielded an AUC of 1.00, confirming flawless class separation and zero false positives or false negatives. As shown in Table 9, the class-wise metrics further validate these findings, with all fault types achieving a true positive rate (TPR) of 1.00 and minimal or zero values for false positive rate (FPR), false negative rate (FNR), and false discovery rate (FDR), except for a slight drop in normal class due to minor misclassifications—demonstrating the model’s reliability and precision across fault categories.

**Figure 13:**
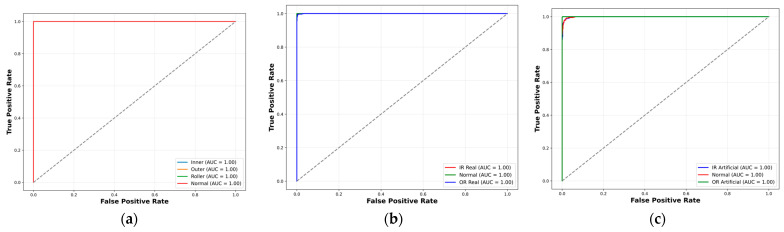
ROC curves of the proposed model on (**a**) UIAI, (**b**) Paderborn real, and (**c**) Paderborn artificial datasets, indicating strong class-wise discrimination performance.

These confidence interval (CI) values reflect a high-performing model with minimal variance, making them statistically realistic and better for bearing fault diagnosis [43]. To evaluate generalization and real-world applicability, the model was further tested on the Paderborn bearing dataset, which includes both real-world and artificially induced fault conditions. On the real dataset, the model achieved an overall accuracy of 97.98 ± 0.18%, with precision, recall, and F1-score all at 98.33 ± ~0.16%, as mentioned in Table 9. Class-wise performance remained high, with perfect classification for normal and OR real samples, and minor misclassifications between IR real and OR real classes. The confusion matrix confirmed 739/774 correct predictions for IR real, 395/395 for normal, and 615/616 for OR real, as shown in Figure 11. As shown in Figure 12, the t-SNE visualizations indicated clear class separation in all three datasets, with minor overlaps observed in the Paderborn real set due to naturally developed faults. Similarly, the ROC curves in Figure 13 reflect strong classification capability, with high AUC values supporting the model’s effectiveness in distinguishing fault types across diverse conditions.

On the artificial Paderborn dataset, the model achieved an even higher accuracy of 98.71 ± 0.15%, with precision, recall, and F1-score values all at 98.67 ± ~0.13%. Only a few misclassifications were observed between IR artificial and normal samples, while OR artificial was classified with 100% accuracy. The ROC curves for all artificial classes once again demonstrated an AUC of 1.00, and t-SNE visualizations in Figure 12 and Figure 13, respectively, revealed distinct, non-overlapping class clusters, further validating the model’s robustness and reliability.

An ablation study was conducted to assess the individual contributions of core components in the proposed model using the UIAI lab dataset. As shown in Table 9, four configurations were tested: no BiLSTM (CWT + Conv1D + MHSA), no MHSA (CWT + Conv1D + BiLSTM), no Conv1D (CWT + MHSA + BiLSTM), and Conv1D only (CWT + Conv1D). The results indicates that excluding BiLSTM led to an average accuracy of 82.12 ± 0.22%, precision of 83.60 ± 0.21%, recall of 82.59 ± 0.20%, and F1-score of 83.09 ± 0.22%. Removing MHSA yielded improved results with 86.50 ± 0.19% accuracy, 87.05 ± 0.17% precision, 87.01 ± 0.19% recall, and 87.07 ± 0.18% F1-score. The best performance among ablation variants was achieved when Conv1D was removed, resulting in 95.05 ± 0.14% accuracy, 96.96 ± 0.13% precision, 96.87 ± 0.15% recall, and 96.91 ± 0.13% F1-score. In contrast, the Conv1D Only configuration produced the poorest performance with 63.68 ± 0.16% accuracy, 62.55 ± 0.18% precision, 62.62 ± 0.17% recall, and 62.43 ± 0.20% F1-score, highlighting the critical contributions of both temporal modeling and attention mechanisms in the proposed framework.

In comparison to recent state-of-the-art models, the proposed method demonstrates notable improvements in both accuracy and robustness. For instance, the model developed by Guanghua Fu et al. [44], which integrates a CNN with BiLSTM and a residual module, employs a dual-path feature extraction strategy. In this framework, BiLSTM captures temporal characteristics from vibration signals, while CNN processes spatial features from time-frequency representations. These complementary features are fused and further refined by a residual module to enhance robustness, particularly under noisy conditions. When this model was applied to the same UIAI Lab dataset, it achieved an overall accuracy of 92.02 ± 0.20%, with precision, recall, and F1-score values of 92.50 ± ~0.22%, respectively, as shown in Table 9. While it outperformed basic CNN and BiLSTM models individually, it still exhibited several misclassifications across fault classes. In contrast, the proposed model not only surpassed Fu et al.’s architecture in all evaluation metrics but also maintained perfect or near-perfect class separation, illustrating its superior capability in handling complex, real-world vibration signals.

Overall, these results highlight the strength of the proposed framework in delivering high-precision and reliable bearing fault diagnosis. Its consistent performance across both controlled and real-world datasets confirms its strong generalization ability and practical relevance for industrial scenarios. The ablation analysis further validates the critical contribution of each architectural component, demonstrating a well-balanced and optimized design. To assess real-time suitability, the model’s latency and complexity were evaluated. It achieved an average inference time (note that the reported inference time refers only to the model’s forward pass and does not include preprocessing steps such as CWT computation) of 0.19 ms per sample measured over 100 runs on an NVIDIA RTX 3060 GPU and contained only 262,308 trainable parameters, with a model size under 1 MB. These results confirm its feasibility for real-time deployment on edge or embedded industrial systems. Moreover, the model effectively tackled key challenges such as nonstationary signal characteristics, class imbalance, and noise interference, making it a robust and scalable candidate for deployment in real-time predictive maintenance and intelligent health monitoring systems.

## 4. Conclusions

This study presents a hybrid deep learning approach for bearing fault diagnosis that integrates continuous wavelet transform (CWT) with an attention-enhanced spatiotemporal feature extraction framework. The model combines multi-head self-attention (MHSA), bidirectional long-short-term memory (BiLSTM), and a 1D convolutional residual network (1D conv ResNet) to effectively capture both spatial and temporal features from nonstationary and nonlinear vibration signals. By leveraging CWT-based time-frequency representations and the complementary strengths of attention and sequential modeling, the approach enabled robust and highly accurate fault classification. Evaluated on the UIAI Lab dataset as well as the real and artificial subsets of the Paderborn dataset, the model consistently achieved near-perfect performance, with precision, recall, and F1-scores close to 1.00. It also demonstrated excellent fault separability in both t-SNE and ROC analyses. The model’s strong generalization capability and resilience to noise affirm its practical suitability for real-time predictive maintenance in industrial environments.

Despite its high accuracy, the model shows slight limitations in distinguishing overlapping fault types. Future work could focus on enhancing class separability using advanced attention mechanisms or multi-scale feature fusion. Additionally, deploying models in real-time environments via edge computing could further improve its practical applicability in dynamic industrial settings.

## Figures and Tables

**Table 1:** Layer-wise architecture of the proposed hybrid model.

Layer Type	Parameters	Activation	Output Shape
Conv2D	(1, 32, 3 × 3)	ReLU	(32, 128, 128)
BatchNorm2D	32	-	(32, 128, 128)
Conv2D	(32, 64, 3 × 3)	ReLU	(64, 128, 128)
BatchNorm2D	64	-	(64, 128, 128)
AdaptiveAvgPool2D	(16, 16)	-	(64, 16, 16)
Flatten		-	(256, 64)
Multi-head attention	4 heads (64 dim)	-	(256, 64)
BiLSTM (2 layers)	Hidden size: 128	-	(256, 256)
Fully connected	1024	ReLU	1024
Fully connected	512	ReLU	512
Fully connected	4	softmax	4

**Table 2:** Key training parameters used for optimizing the proposed deep learning model.

Parameter	Value
Optimizer	Adam
Learning rate	0.0005
Scheduler	StepLR (step_size = 10, gamma = 0.5)
Loss function	CrossEntropyLoss
Batch size	32
Epochs	40

**Table 3:** Specifications of the data acquisition system.

Device	Specification	Value
Vibration sensor (PCB-622B01)	Measurement range	±490 m/s^2^
	Frequency	0.2–15,000 Hz
	Sensitivity	100 mV/g
AE sensor (R151-AST)	Operating range	50–400 kHz
	Resonant frequency	150 kHz
	Peak sensitivity	−22 dB
DAQ (NI 9234)	Dynamic range	102 dB
	Resolution	24-bit
	Operating temperature	−40 °C–70 °C

**Table 4:** Data acquisition summary.

Testing Condition	Samples Count	Sampling Rate (KHz)	Time (min)
IRF	370	25	5
NC	344	25	5
ORF	347	25	5
RF	309	25	5

**Table 5:** Operating conditions.

S. No	Rotational Speed (rpm)	Load Torque (Nm)	Radial Force (N)
0	1500	0.7	1000
1	900	0.7	1000
2	1500	0.1	1000
3	1500	0.7	400

**Table 6:** Details of bearing codes used in Case 1.

Bearing State	Bearing Code
Normal	K001, K002, K003, K004, K005, K006
Artificial IR fault	KI01, KI03, KI05, KI07, KI08
Artificial OR fault	KA01, KA03, KA05, KA06, KA07, KA09

**Table 7:** Details of bearing codes used in Case 2.

Bearing State	Bearing Code
Normal	K001, K002, K003, K004, K005, K006
Real IR fault	KI04, KI14, KI16, KI17, KI18, KI21
Real OR fault	KA04, KA15, KA16, KA22, KA30

**Table 8:** Proposed model evaluation of performance metrics on diverse datasets and comparison with Fu et al. and ablation studies.

Performance Metrics	Proposed			Fu et al [44].	Ablation Study (UIAI Dataset)			
Datasets	UIAI	Real Paderborn	Artificial Paderborn	UIAI	No BiLSTM	No MHSA	No Conv1D	Conv1D Only
**Accuracy (%)**	99.27 ± 0.12	97.98 ± 0.18	98.71 ± 0.15	92.02 ± 0.20	82.12 ± 0.22	86.50 ± 0.19	95.05 ± 0.14	63.68 ± 0.16
**Precision (%)**	99.25 ± 0.11	98.33 ± 0.16	98.67 ± 0.14	92.50 ± 0.21	83.60 ± 0.21	87.05 ± 0.17	96.96 ± 0.13	62.55 ± 0.18
**F1 Score (%)**	99.25 ± 0.10	98.33 ± 0.15	98.67 ± 0.13	92.50 ± 0.22	83.09 ± 0.22	87.07 ± 0.18	96.91 ± 0.13	62.43 ± 0.20
**Recall (%)**	99.25 ± 0.13	98.33 ± 0.17	98.67 ± 0.12	92.50 ± 0.23	82.59 ± 0.20	87.01 ± 0.19	96.87 ± 0.15	62.62 ± 0.17

**Table 9:** Class-wise TPR, FPR, FNR, and FDR showing the model’s accuracy and error distribution across fault types.

Class	True Positive Rate (TPR)	False Positive Rate (FPR)	False Negative Rate (FNR)	False Discovery Rate (FDR)
Inner	1.0000	0.0099	0.0000	0.0274
Outer	1.0000	0.0000	0.0000	0.0000
Roller	1.0000	0.0000	0.0000	0.0000
Normal	0.9730	0.0000	0.0270	0.0000

## Data Availability

The raw data supporting the conclusions of this article will be made available by the authors on request.

## Nomenclature

BFD	bearing fault diagnosis
CWT	continuous wavelet transform
CNN	convolutional neural network
BiLSTM	bidirectional long short-term memory
ReLU	rectified linear unit
TPR	true positive rate
ANN	artificial neural network
UIAI	Ulsan Industrial Artificial Intelligence
